# Cross-sectional assessment of cardiovascular risk factors in patients with knee osteoarthritis

**DOI:** 10.12688/f1000research.27744.2

**Published:** 2021-11-25

**Authors:** Sagar Goel, Surendra Umesh Kamath, Rajendra Annappa, Sunil Lakshmipura Krishnamurthy, Manesh Jain, Samarth Thakkar, Lulu Damsas, Sayak Banerjee, Prajwal Madapura Divakar

**Affiliations:** 1 Department of Orthopedics, Kasturba Medical College, Mangalore, Manipal Academy Of Higher Education, Manipal, Karnataka, 576104, India

**Keywords:** Osteoarthritis, Cardiovascular disease, Cardiovascular risk factors, JBS3 risk, Hypertension, Diabetes Mellitus, Smoking, Age, Sex, SES, Heart Age, Life Expectancy, Kellgren Lawrence

## Abstract

**Background:** Osteoarthritis (OA) and cardiovascular disease (CVD) are prevalent in India. However, there is dearth of literature among Indians studying the relationship between the two. This study was carried out to assess various cardiovascular (CV) risk factors in patients with knee OA with an objective to investigate their association, screening and management.

**Methods: **In total, 225 patients were included in this cross-sectional study. Participants were diagnosed with knee OA on the basis of the Kellgren and Lawrence (K-L) classification of their radiograph. Participants were also assessed for CV risk factors (age, body mass index, systolic blood pressure, diabetes mellitus, total cholesterol, high-density lipoprotein, smoking) with the help of the Joint British Society QRisk3 calculator (JBS3) a comprehensive risk score calculator as well as a screening tool, which produces three more variables, namely 10-years risk of developing CVD, physiological heart age and life expectancy. Chi Square, Fishers exact test and one-way ANOVA tests were used to compare the categorical and quantitative variables, respectively. Pearson’s correlation coefficient was used to assess the relationship between CV risk factors and knee OA. Multiple regression analysis was done to adjust the multiple con-founders and determine their significance.

**Results:** Patients with severe knee OA had a statistically significantly higher prevalence of CV risk factors (p<0.05). Grade 4 knee OA patients were found to have a mean JBS3 risk of 38%, heart age of 82 years and life expectancy of 77 years as compared to grade 2 patients who had a mean JBS3 risk of 11%, heart age of 63 years and life expectancy of 82 years.

**Conclusions: **Our study concluded that there is a strong positive correlation between knee OA and CVD, with CV risk score being directly proportional to the severity of OA.

## Introduction

Osteoarthritis (OA), a degenerative joint disease, is the sixth most predominant cause of disability around the world and a well-established cause of restricted activity, disability, and low quality of life. ^1^ Its prevalence, currently at 3.5% of the world’s total population and about 35% in adults older than 60 years ^2^, is on the rise. OA has become an important public health issue and is burdensome for both personal health and social well-being. ^2^ Both developed and developing nations see it as a major liability on their health care sector with it having severe social and economic impacts. ^1^

OA is associated with knee pain, functional limitation and deformity and it incurs articular cartilage degeneration, osteophyte formation in joints, reduced joint space and subchondral sclerosis. ^3^ The etiology of OA is idiopathic, yet age, genetics, obesity, menopause, hypertension, and diabetes mellitus have been found to be major contributors. ^4^ Some of these risk factors that are directly involved and others indirectly involved in the etiopathogenesis of OA are also seen to increase the risk of cardiovascular diseases (CVDs), namely congestive heart failure (CHF), ischemic heart disease (IHD), transient ischemic attacks (TIA) and stroke. ^4^ This group of cardiovascular diseases are also the leading cause of mortality and morbidity worldwide. ^5^ Therefore, it becomes important to identify the various cardiovascular risk factors like age, gender, obesity, hypertension, cholesterol, sedentary lifestyle, smoking and nutrition at the earliest opportunity through various screening methods, especially in patients with OA. ^3^

A possible association between OA and cardiovascular risk factors is evident by Dr J.S. Lawrence work at Leigh where he obtained above average levels of serum cholesterol, especially in women with osteoarthritis of hands. ^3^ Singh *et al*. also found a high prevalence of cardiovascular risk factors in adults with knee OA. ^5^ Recently, a new classification for phenotyping of OA, which includes metabolic syndrome, aging, and posttraumatic arthritis, has been developed. Obesity is a well-known contributor for metabolic syndrome. ^6^ Observational studies have shown that hypertension is an independent risk factor for knee OA. ^6^^,^^7^ Indirectly, physical inactivity, due to debilitating joint pain, also makes patients more prone to CVD. ^7^

Given that OA and CVD are common conditions in the elderly, a thorough knowledge of the relationship between OA and CVD could help further our understanding of potential biological and behavioral mechanisms, which could in turn help in making informed decisions regarding further management of OA. There is a dearth of literature relating knee OA and cardiovascular risk factors in the Indian population, hence we wanted to study this association among Indian participants. The objectives of the study were (i) to screen patients diagnosed with knee OA for cardiovascular risk factors who don’t have a history of cardiovascular diseases; (ii) to find out the risk of developing a cardiovascular disease in next 10 years in patients suffering from knee OA using a simple standardized risk score calculator; and (iii) to correlate CVD risk with severity of knee OA.

## Methodology

### Ethics statement

The research protocol with the informed consent form was approved in October 2018 by the Institutional Ethics Committee of Kasturba Medical College, Mangaluru (reference number IEC KMC MLR 10-18/363). Participants provided written informed consent to participate. In addition, case records of patients diagnosed with knee OA between January 2017 to September 2018 were studied. Permission to access the previous records was obtained from the medical suprintendant of the hospitals associated with Kasturba Medical College, Mangalore through the proper channel including head of department of orthopedics and Institutional Ethics committee. Confidentiality of the participants was maintained by concealing their name and identity. Each participant was given a unique serial number. The patients were not charged extra for any test or investigation other than their routine healthcare charges.

### Study design

This cross-sectional study was done in the tertiary care centres associated with Kasturba Medical College, Mangalore, India. The study was carried out between October 2018 and September 2020.

### Study participants

Patients diagnosed with knee osteoarthritis (Kellgren and Lawrence (KL) grade ≥2 on knee X-ray ^8^) and ≥50 years old ^9^ were invited to take part. The KL grading system is as follows:

1. Grade 0 (none): the definite absence of x-ray changes of osteoarthritis
2. Grade 1 (doubtful): doubtful joint space narrowing and possible osteophyte lipping
3. Grade 2 (minimal): definite osteophyte and possible joint space narrowing
4. Grade 3 (moderate): moderate multiple osteophytes, definite narrowing of joint space, and some sclerosis and possible deformity of bone ends.
5. Grade 4 (severe): large osteophytes, marked narrowing of joint space, severe sclerosis and definite deformity of bone ends.

Exclusion criteria were patients who refused to give consent, patients who were suffering from secondary knee OA, and patients who were known cases of coronary artery disease.

The sample size was calculated to be 187 using the Cochran’s formula ^10^:

$$n={\frac{p*q*Z\propto }{d^{2}}}^{2}$$

where, Z∝ = 1.96 at 95% confidence level

p (estimated proportion of an attribute that is present in the population) = 34% with respect to study by Kim HS *et al.* ^11^ This is comparable to the prevalence of knee OA found in Indian population. In a community based cross sectional study Chandra Prakash Pal et al found overall prevalence of knee OA to be 28.7%. ^12^ Along similar lines,another community based cross sectional study by Jayaseelam  Venkatachalam et al showed that out of a total of 1986 adults 27.1% had knee OA. ^13^

q = 66% (q = 100-p)

d (desired precision) = 20% of p at 80% power

With 95% confidence level and 80% power. Though the sample size was calculated to be 187, we collected data from 225 patients due to additional time and resources.

### Sampling

This study employed convenience sampling. Patients aged more than 50 years presenting to the orthopedics out-patient department with complains of knee pain between October 2018 and september 2019 were recruited for the study. Also, case records of patients diagnosed with knee OA between January 2017 to September 2018 were studied. No prior randomization was done.

### Variables

Patient demographics such as age, gender, weight, height, body mass index (BMI), socioeconomic status and their smoking habits were recorded. Weight bearing knee radiographs in anterior, posterior and lateral views were done. Those with knee OA of K-L grade of 2 or more were included in the study. Serum total cholesterol and high-density lipoprotein (HDL) was determined. History of ongoing treatment for hypertension, diabetes, RA and CVD in relatives before age of 60 years was gathered. All these variables were added in the JBS3 risk score calculator which then produced three more variables; physiological heart age, life expectancy, and JBS3 risk of developing CVD in the next 10 years.

JBS3 risk score calculator was devised by joint British society for general practitioners to help guide their work with patients, in preventing CVD. A major role of the JBS3 risk score calculator is the idea of estimating CVD risk over a lifetime. Patients can also be screened for cardiovascular risk factors that they are unaware of.

### Procedure

Case records of patients aged 50 years and above, diagnosed with knee osteoarthritis, between January 2017 and September 2018, and patients coming to the Out Patient Department (OPD) from October 2018 to September 2019 were studied. Patients were briefed about the aims and implications of the study, and consenting patients were recruited into the study.

Each patient was given a unique serial number and a detailed history and examination were done by the principle investigator of the study. History of presenting illness along with past, treatment and family history were gathered. Examination included general physical examination along with cardiovascular systemic examination and local knee examination. Orthopedic evaluation was done using K-L grading ^9^ and CV risk was determined using the JBS3 CV risk score calculator. ^14^ Fasting blood sample was collected to know the levels of their serum total cholesterol and serum high density lipoprotein. The standard procedure of phlebotomy was followed. 3ml of blood was drawn from the patient and collected in plain vacutainer for serum analysis of total cholesterol and serum high density lipoprotein. The sample was sent to Biochemistry laboratory where they analysed using enzymatic colorimetric method using enzyme cholesterol esterase and peroxidase for total cholesterol and polymer polyanion method for HDL. Patient details and blood test results were uploaded to the unique profile created for each one of them in the mobile application based JBS3 CV risk score calculator ^14^ which then predicted physiological heart age, life expectancy, and JBS3 risk of developing CVD in the next 10 years.

### Statistical analysis

The Statistical Package for the Social Sciences ( SPSS) version 20 was used to do the statistical analysis. The categorical variables like age, gender, smoking habits, ongoing treatment for hypertension, diabetes, history of CVD in relatives before age of 60 years and history of rheumatoid arthritis (RA) were compared with K-L grade of knee OA using chi square test. Socioeconomic status and history of RA were compared with K-L grades using Fishers exact test. The quantitative variables like BMI, total cholesterol, HDL, physiological heart age, life expectancy and 10 years risk of developing CVD were compared with K-L grades using analysis of variance (one-way ANOVA) test. A p value of <.05 was considered significant and less than 0.01 was considered highly significant.

Post hoc analysis was done to compare multiple group means. The mean heart age and mean physiological heart age among the three K-L grades of knee OA were compared using paired t test. A p value <.05 was considered significant. Pearson’s correlation coefficient was employed to determine the correlation between the K-L grade of knee OA and quantitative variables like serum total cholesterol (TC), HDL, BMI, SBP, physiological heart age, life expectancy and 10 years risk of developing CVD. Receiver operating characteristic (ROC) curve was used to show in a graphical way the connection/trade-off between clinical sensitivity and specificity for 10 years risk of developing CVD in the study population.

## Results

**Figure 1.**
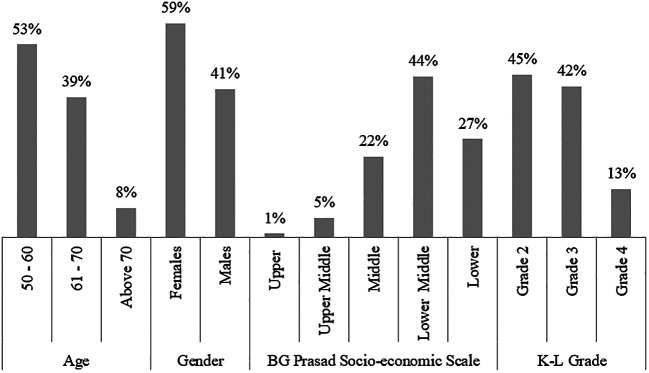
Age, Gender, BG Prasad socioeconomic scale, KL grade, BMI and history of smoking distribution of the study population.

A total of 225 patients visiting the orthopedics OPD with complaints of knee pain took part in the study. ^31^ All participants were over 50 years of age with more than half (53%) of them between the age of 50-60 years ( Figure 1). The majority of these patients were females (59%) ( Figure 1). As per the BG Prasad socioeconomic scale (SES), ^15^ the majority of them belonged to either the middle or lower socio-economic group ( Figure 1).

**Figure 2.**
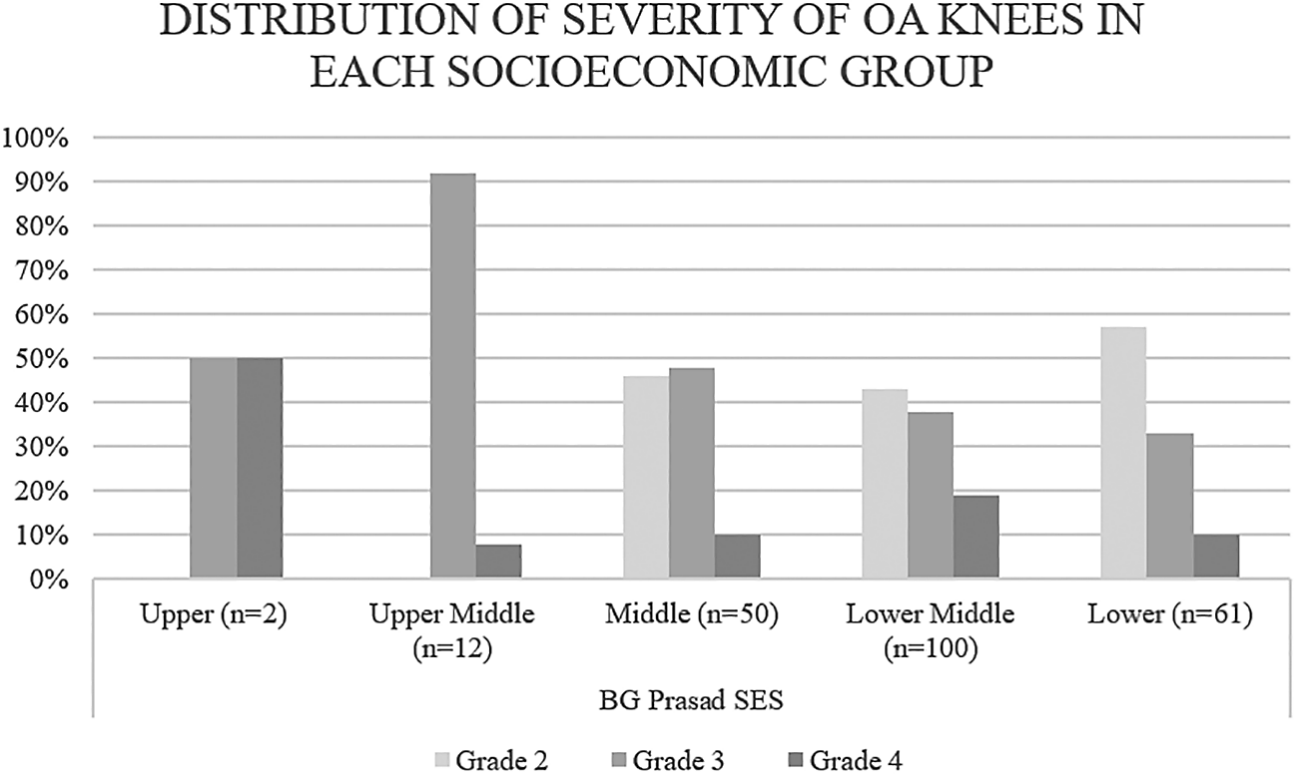
Distribution of knee osteoarthritis (OA) severity by socioeconomic group. Higher socioeconomic groups had a higher proportion of patients with severe grade knee OA.

Grade 2 K-L classification of knee OA was found to be the most common (45%) among the study population. Higher grades of knee OA, i.e. 3 and 4, were found to be 42% and 13%, respectively ( Figure 1). Overall, 57% of the patients belonging to the lower socio-economic class as per the BG Prasad SES had knee OA grade 2, 33% of them had grade 3 and only 10% had grade 4 OA. Among the lower middle and middle-class groups, a decrease in the percentage of patients with grade 2 and increase in percentage of patients with grade 3 and 4 was found as socioeconomic status increased. The same trend was observed, not strictly though, as we go further up the SES. Upper middle and upper-class patients had either grade 3 or 4 knee OA ( Figure 2).

The Fishers exact test was used to find out the statistical significance of the relationship between socioeconomic status and knee OA grade, which was found to be highly significant (p value<.01). Chi square test, when applied to compare knee OA grade with age {X ^2^(4, 225) = 3.801, p = .434} or gender {X ^2^(2, 225) = 1.016, p = .602}, was not found to be statistically significant. A one-way ANOVA test was applied to K-L grade of knee OA with BMI and found to be highly statistically significant {F(4,225) = 60.652, p < .01}. An increasing trend in the BMI of patients was noted with a higher grade of knee OA, e.g. 60% of grade 2 knee OA patients had a BMI of less than 25 as compared to 40% in grade 3 and only 7% in grade 4. In addition, 57% of patients with grade 4 knee OA had a BMI of more than 30 as compared to 13% in grade 3 and only 4% in grade 2 ( Table 1).

**Table 1.** Results of all of the qualitative variables against each of the grade of severity of knee OA ( K-L Grade) and p value as measured by chi square/Fischer s exact test. Count = number of study samples in each K-L grade with respect to the corresponding variable; Total = number of study samples in each K-L grade; Column N% = Count/Total; Row N% = Count/number of study samples in a subgroup of a variable. p value <.05 is considered significant, p value of <.01 is considered highly significant.

		Severity of knee OA (K-L Grade)									Chi square/Fishers exact test p value
		Grade 2			Grade 3			Grade 4			
		Count	Column N %	Row N %	Count	Column N %	Row N %	Count	Column N %	Row N %	
BMI	< 25	60	59%	60%	38	40%	38%	2	7%	2%	.000
	25 - 30	37	37%	40%	44	47%	48%	11	37%	12%	
	> 30	4	4%	12%	12	13%	36%	17	57%	52%	
	Total	101	100%	45%	94	100%	42%	30	100%	13%	
History of smoking	No	85	84%	46%	76	81%	41%	23	77%	13%	.617
	Yes	16	16%	39%	18	19%	44%	7	23%	17%	
	Total	101	100%	45%	94	100%	42%	30	100%	13%	
History of antihypertensive treatment	No	83	82%	70%	27	29%	23%	8	27%	7%	.000
	Yes	18	18%	17%	67	71%	63%	22	73%	21%	
	Total	101	100%	45%	94	100%	42%	30	100%	13%	
History of Diabetes Mellitus	No	97	96%	58%	56	60%	34%	14	47%	8%	.000
	Yes	4	4%	7%	38	40%	66%	16	53%	28%	
	Total	101	100%	45%	94	100%	42%	30	100%	13%	
H/O of CVD in near relative of <60 years of age	No	96	95%	51%	78	83%	41%	16	53%	8%	.000
	Yes	5	5%	14%	16	17%	46%	14	47%	40%	
	Total	101	100%	45%	94	100%	42%	30	100%	13%	
History of Rheumatoid arthritis	No	101	100%	47%	85	90%	40%	28	93%	13%	.007
	Yes	0	0%	0%	9	10%	82%	2	7%	18%	
	Total	101	100%	45%	94	100%	42%	30	100%	13%	
JBS3 (%)	0 - 10	56	55%	76%	15	16%	20%	3	10%	4%	.000
	11 - 25	41	41%	44%	45	48%	48%	8	27%	9%	
	> 25	4	4%	7%	34	36%	60%	19	63%	33%	
	Total	101	100%	45%	94	100%	42%	30	100%	13%	

The mean BMI of patients with grade 2 knee OA was 24.82, grade 3 was 26.39 and grade 4 was 30.96, with each knee OA grade showing statistically significant data when compared with the BMI of patients ( Table 2). In post hoc analysis using the Bonferroni test with BMI as the dependent variable, it was found that multiple comparisons of each of grade of knee OA with the other were found to be statistically significant, as depicted in Table 3.

**Table 2.** Total count, mean, standard deviation, ANOVA p value and significance of all of the quantitative variables against each of the grade of severity of knee OA (K-L Grade). p value <.05 is considered significant, p value <.01 is considered highly significant.

	Severity of knee OA (K-L Grade)	Count N	Mean	Standard deviation	95% Confidence interval for mean		ANOVA p value	Inference
					Lower bound	Upper bound		
BMI	2	101	24.8289	2.79067	24.2780	25.3798	.000	Highly significant
	3	94	26.3934	3.60523	25.6550	27.1318		
	4	30	30.9680	4.02460	29.4652	32.4708		
Mean systolic BP (mm Hg)	2	101	128.57	14.847	125.64	131.51	.000	Highly significant
	3	94	139.40	17.519	135.82	142.99		
	4	30	150.07	14.607	144.61	155.52		
Total cholesterol (mg/dl)	2	101	188.34	32.987	181.82	194.85	.000	Highly significant
	3	94	213.66	31.311	207.25	220.07		
	4	30	234.90	36.159	221.40	248.40		
High density lipoprotein (mg/dl)	2	101	53.74	7.152	52.33	55.15	.013	Significant
	3	94	51.99	7.444	50.46	53.51		
	4	30	49.30	8.014	46.31	52.29		
Mean age	2	101	59.72	6.763	58.39	61.06	.035	Significant
	3	94	61.71	6.558	60.37	63.06		
	4	30	62.80	7.194	60.11	65.49		
Mean physiological age	2	101	63.06	9.330	61.22	64.90	.000	Highly significant
	3	94	74.77	11.671	72.38	77.16		
	4	30	82.80	12.007	78.32	87.28		
Life expectancy	2	101	82.27	4.032	81.47	83.06	.000	Highly significant
	3	94	79.71	4.357	78.82	80.61		
	4	30	77.33	5.536	75.27	79.40		
JBS3 risk score (%)	2	101	11.18	6.560	9.88	12.47	.000	Highly significant
	3	94	23.19	14.433	20.24	26.15		
	4	30	37.86	22.043	29.63	46.09		

**Table 3.** Post hoc analysis of each of the variables using Bonferroni method and p value. p value <.05 is considered significant, p value <.01 is considered highly significant.

Bonferroni dependent variable	Comparisons between each grade of severity of knee OA		Bonferroni test p value	Inference
BMI	2	3	.004	Highly significant
		4	.000	Highly significant
	3	4	.000	Highly significant
Mean systolic BP	2	3	.000	Highly significant
		4	.000	Highly significant
	3	4	.005	Highly significant
Total cholesterol	2	3	.000	Highly significant
		4	.000	Highly significant
	3	4	.007	Highly significant
High density lipoprotein	2	3	.299	Not significant
		4	.013	Significant
	3	4	.252	Not significant
Mean age	2	3	.121	Not significant
		4	.039	Significant
	3	4	1.00	Not significant
Mean physiological age	2	3	.000	Highly significant
		4	.000	Highly significant
	3	4	.001	Highly significant
Life expectancy	2	3	.000	Highly significant
		4	.000	Highly significant
	3	4	.031	Significant
JBS3 risk score	2	3	.000	Highly significant
		4	.000	Highly significant
	3	4	.000	Highly significant

Of the 225 participants, 18.2% (n = 41) were smokers. All the smokers were male, and 39% of them had grade 2 knee OA, 44% of them had grade 3 and 17% of them had grade 4. However, this data was not statistically significant on chi square test {X ^2^(2, 225) = 0.964, p = .617} ( Table 1).

The mean systolic blood pressure (SBP) of participants was 128mmhg, 139mmhg and 150mmhg for grade 2, grade 3 and grade 4 knee OA, respectively. Table 2 shows the representation of mean SBP for each K-L grade. On comparing each grade with each other using post hoc analysis with SBP as the Bonferroni dependent variable, the comparisons came out to be highly significant ( Table 3).

Of the 225 participants, 88 had a SBP reading of 140 or more, out of which 28 (33%) were not taking any antihypertensive medication. In total, 25.8% of the entire sample had a history of diabetes mellitus (DM), which is much lower than more than half (53%) of patients with grade 4 knee OA. Of grade 3 patients, 40% had DM, yet of grade 2, only 4% had DM. This was found to be statistically significant {X ^2^(2, 225) = 47.574, p < .01} as per the chi square test ( Table 1).

Similar trends were seen in the history of CVD among the relatives of the study population. A total of 35 (15.5%) patients gave a history of their relatives suffering from CVD in the past. Compared by grade, 46.7% of the patients with grade 4 knee OA gave a positive relative CVD history as compared to only 17% and 5% of the patients with grade 3 and 2 knee OA, respectively ( Table 1). The chi square test for this difference was statistically significant {X ^2^(2, 225) = 30.907, p < .01}.

None of the patients in the study population suffered atrial fibrillation (AF) or gave a history of chronic kidney disease in the past. Only 11 patients gave a history of rheumatoid arthritis which was found to be statistically significant by Fisher exact test (p < .01) ( Table 1).

Serum total cholesterol (TC) and high-density lipoprotein (HDL) were tested for all the patients. Table 2 shows the mean levels of TC and HDL with respect to each of the KL grades of knee OA. An increase in mean value of TC and decrease in mean value of HDL were noted as K-L grades of knee OA increase. Patients with grade 2 knee OA had a mean TC value of 188.34 and mean HDL value of 53.74. Similarly, patients with grade 3 knee OA had a mean TC and HDL value of 213.66 and 51.99, respectively. Patients with grade 4 knee OA had a mean TC and HDL value of 234.90 and 49.30, respectively ( Table 2).

In post hoc analysis using the Bonferroni test with TC as the dependent variable, it was found that multiple comparisons of each of grade of knee OA with the other were found to be highly statistically significant ( Table 3). The same was not true, however, with HDL, which showed statistically significant data only when K-L grade 2 was compared to grade 4 knee OA ( Table 3).

**Figure 3.**
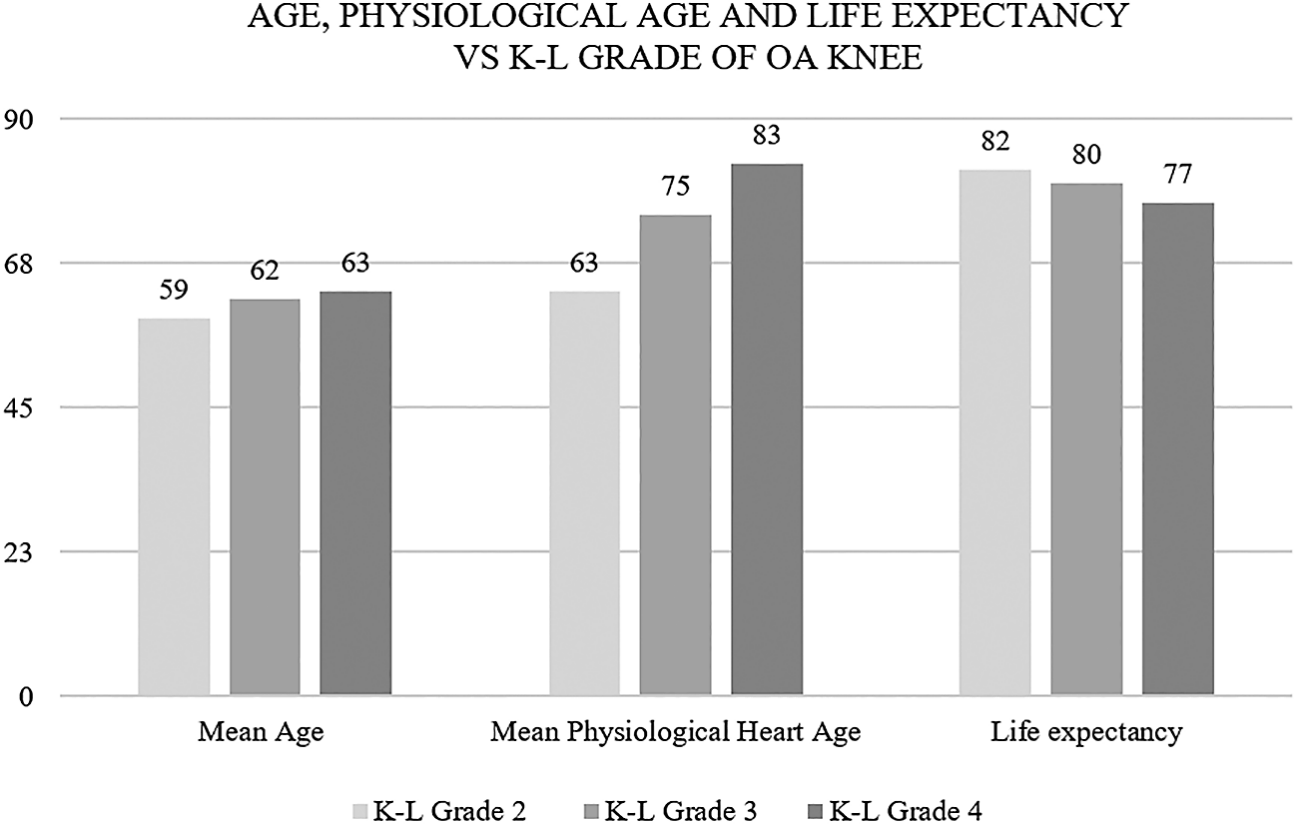
As we go up the K-L grade of knee OA, the physiological heart age went up and the life expectancy came down.

This JBS3 risk score calculator gave us three parameters about the cardiovascular condition of the heart, i.e. physiological heart age, 10-year risk in percentage of developing a CVD and life expectancy provided any other cause of death is ruled out. Statistically significant results were obtained when studying the severity of knee OA with heart age and life expectancy. Physiological heart age came out lesser but life expectancy higher for patients with a lower grade of knee OA. As we go up the K-L grade of knee OA, the physiological heart age went up and the life expectancy came down ( Figure 3).

The mean physiological heart age was found to be 70 years; 10 years older than the mean chronological age (60) of the study population.

The JBS3 risk score also increased with the increase in the K-L grade of knee OA. Patients with grade 2 knee OA had a mean risk of around 11% of developing a CVD in the next 10 years and this increased with K-L grades, i.e. there was a 23% risk among the patients with grade 3 knee OA and 38% risk among those with grade 4 knee OA ( Table 2). Chi square test found this to be statistically significant {X ^2^(4,225) = 70.776, p < .01}.

**Figure 4.**
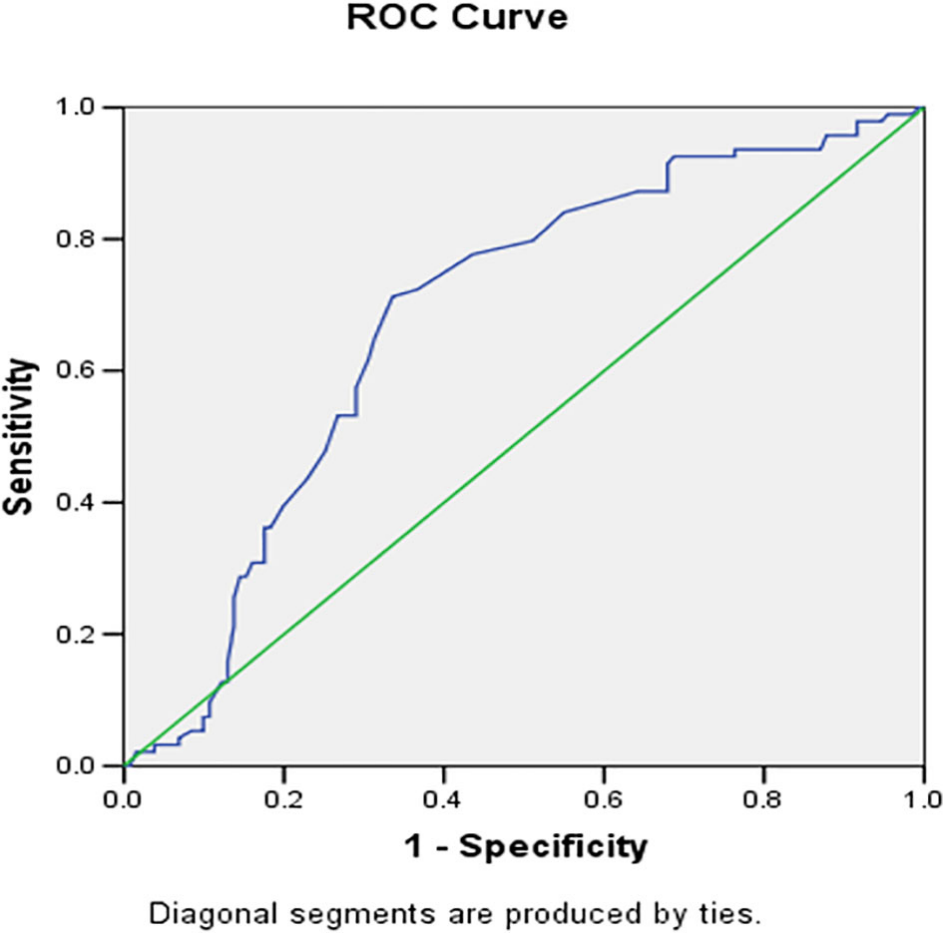
ROC analysis for the JBS 3 risk score showed that a patient with K-L grade 4 of knee OA is more likely to be running a 10 years risk of developing CVD at minimum 14% with sensitivity of 72.3% and specificity of 66%.

**Figure 5.**
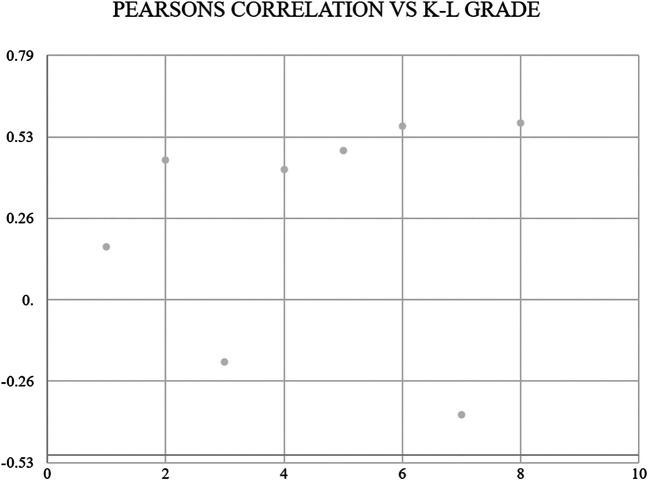
A positive Pearson's correlation was seen between K-L grade and JBS 3 risk, age, TC, SBP, BMI, and physiological heart age. A negative correlation was seen with HDL and life expectancy.

The Bonferroni post hoc analysis of K-L grade with the JBS3 risk score as the dependent variable showed multiple comparisons of each K-L grade with the others as statistically significant ( Table 3). The ROC curve showing area under the curve of sensitivity vs specificity of grade 4 vs 2 and 3 for risk score is depicted in Figure 4. According to ROC analysis for the JBS3 risk score, it was determined that patients with K-L grade 4 knee OA were more likely to run a 10-year risk of developing CVD at minimum 14% with sensitivity of 72.3% and specificity of 66%. Pearson’s correlation was derived, showing a positive correlation between K-L grade and JBS3 risk score with a correlation factor of 0.57 (p < .01). Similarly, a positive correlation was seen with age, TC, SBP, BMI, and physiological heart age. A negative correlation is seen with HDL and life expectancy ( Figure 5). On analyzing the data using multiple regression with dependent variable as K-L Grade of knee OA ( Table 4), we found that variables like history of hypertension treatment, diabetes mellitus, serum levels of total cholesterol and body mass index were found to be significant, rest all variables were found to be not significant.

**Table 4.** Multiple regression analysis with K-L Grade of knee OA as dependent variable.

Variable	Standardized coefficients (Beta value)	p value	Inference
History of antihypertensive treatment	.262	.000	Highly significant
History of Diabetes Mellitus	.115	.043	Significant
H/O of CVD in near relative of <60 years of age	.094	.079	Not significant
History of Rheumatoid arthritis	.084	.095	Not significant
BMI	.283	.000	Highly significant
Total cholesterol	.283	.000	Highly significant
High density lipoprotein	−.067	.168	Not significant
Mean systolic BP	.051	.381	Not significant

## Discussion

Osteoarthritis is a common disease in the elderly population, with its higher prevalence in the 50+ year age group well documented in the literature. ^1^^-^^3^ Many studies have been done in European and American populations to measure the prevalence of OA, but there is a scarcity of studies in Asian populations. One study done by Chandra Prakash Pal *et al.* on the epidemiology of knee OA in India showed a prevalence of 28.7% in the study population. ^12^ Another study in Korea showed increased prevalence with increasing age. ^11^

The current study’s entire sample of 225 patients were above the age of 50 years. Higher prevalence of knee OA in the 50-60-year age group was seen but with no statistical significance when compared with groups of higher ages. This could be attributed to the fact that this study did not include the entire population of the given area but rather only the patients coming to orthopedic OPD with pain in the knee joint. Women are more likely to have knee pain above the age of 50 years ^9^ and with no surprise our study also had women forming almost two thirds (60%) of the sample size. A cross-sectional study done in Korea among their population found a higher prevalence of OA in women (45%) as compared to men (25%). ^11^ The estimated global prevalence of OA is around 10% in men and 20% in women. ^3^^,^^5^^,^^11^^,^^12^^,^^16^

American and European studies have found OA to be a disease of the upper class. ^16^ The COPCORD study from Bangladesh also showed the same. ^17^ On the contrary, a study conducted in south Delhi found the prevalence of OA was higher in perimenopausal women of lower classes than upper classes. ^18^ Our study had higher numbers of patients belonging to lower and lower middle classes but showed increased severity in upper class people. Participants of lower socioeconomic status mostly had grade 2 knee OA but upper classes had the more severe grades of 3 or 4 as per K-L grading. A study in Japan revealed a higher prevalence of OA from mountainous rather than rural or urban regions, attributing the effect of activity leading to wear and tear of joints. ^19^ Although increased activity causes more wear and tear of knee joints, there are other modifiable risk factors that also in some way directly or indirectly affect the knee joint, e.g. obesity, sedentary lifestyle, hypertension, hypercholesterolemia, smoking, diabetes mellitus and other cardiovascular risk factors. ^20^^,^^21^ In the current study, BMI was found to be higher in groups with severe grades of knee OA as compared to milder grades, and there was a statistically significant association found between BMI and K-L grade of knee OA. Upper class people tend to have more modifiable risk factors, ^18^ which is quite evident in the current study, showing increased prevalence of these modifiable risk factors (BMI, TC, HDL, SBP, DM) in these groups.

Other factors like the role of systemic inflammation in the pathomechanisms of knee OA have been long studied ^22^; however, there have been some contradictory results. Atherosclerosis, hypertension, diabetes and hypercholesterolemia cause systemic inflammation in the body, which can lead to CVD. ^23^ Their association with knee OA has been studied by many doctors and researchers who believe that there must be some unknown relationship either directly or indirectly between CV risk factors and knee OA as they both involve systemic inflammation in the body. This belief of some sort of relationship between CV risk factors and knee OA is based on past observational studies that showed prevalence of severe grade OA in patients with CVD and vice versa. Louati K *et al.* in their meta-analysis of 299 publications found a high prevalence of OA in patients with DM. ^6^ They also emphasized that metabolic OA phenotypes needs to be studied further. Huajun Wang *et al.*, ^24^ in their attempt to explore the correlation between metabolic syndrome and knee OA using meta-analysis of eight studies, found a significant odds ratio (OR) even after adjusting for many risk factors. The aforementioned Korean study displayed a significantly higher knee OA prevalence in patients with hypertension and impaired glucose tolerance. ^11^ Veronese *et al.* also found an association between knee OA and CVD, studying 2158 elderly participants without CVD and finding that OA at baseline was associated with subsequent incident CVD. ^23^^,^^25^ In the current study, the mean SBP was significantly higher in patients with K-L grade 3 and 4 of knee OA. The mean SBP was 128 mmhg for K-L grade 2, 139 mmhg for K-L grade 3 and 150 mmhg for K-L grade 4. The chi square test found the differences to be statistically significant and post hoc analysis showed them to be highly significant with each increasing grade. Of the total 225 patients, 88 had a BP reading equal to or higher than 140 mmhg, most of which had a severe knee OA. Similar trends were seen while studying the prevalence of DM in these patients. In in the total sample, 25.8% patients had DM, yet the largest percentage (53%) was seen in patients with K-L grade 4 knee OA, showing the risk of DM increases with severity of knee OA, as found by Louati K *et al.* ^6^ Hypercholesterolemia was also studied here, being another systemic inflammatory marker. It showed a statistically significant relationship with K-L grades of knee OA; mean serum TC was found to be higher in patients with severe grades of knee OA, whilst mean serum HDL was found to be lower in patients with severe grades of OA.

Systolic BP, DM and hypercholesterolemia are three of the major contributors to CVD. ^11^ As per the results of this study it would be safe to say that patients with severe knee OA have a higher risk of developing a CVD in the future. On the contrary, Hoeven *et al*. ^26^ believe that OA-related disability and not OA predicts CVD. They postulated that neither clinical nor radiological knee OA predicts a CV event in the future, rather it’s the disability, independent of OA, that predicts the OA. This is along the lines of belief of many other researchers who postulate that OA doesn’t directly lead to a CVD, rather it renders a patient physically more inactive and disabled than those without OA, which ultimately increases the CV risk factors. Whether OA causes CVD directly or indirectly, it is hard to ignore the fact that they often exist co-dependently in a patient’s body. Assessment of a patient with knee OA for CV risk factors would be of no harm and rather provide the doctor a systemic approach to the patient’s morbid condition and also make the patient aware about their unknown CV risk factors and risk of developing a CVD. Assessment of CV risk factors in patients with OA has been a topic of research in Korea. Jeong *et al.* studied data from the Korean National Health Survey and Nutrition Examination Survey (KNHANES) and found a higher prevalence of hypertension, DM, dyslipidemia, angina and myocardial infarction in patients with OA compared with healthy individuals. ^27^

Contrary to our assumption, smoking was not found to have any statistically significant effect on the K-L grade of knee OA. However, it has a role to play in determining the increased risk of CVD in these patients. ^28^ Other variables such as atrial fibrillation, chronic kidney disease and rheumatoid arthritis were not found to be prevalent in our study population, preventing us from analyzing any relationship between them and knee OA.

This study has attempted to collaborate the basic mechanical, causal and shared risk factors (age, obesity, gender) between knee OA and CVD. It has also tried to study various other CV risk factors (hypertension, DM, dyslipidemia, smoking, family history) in patients with OA. Ho Sun Kim *et al.* ^11^ used the Framingham risk score (FRS) in south Koreans to study the association between OA and CV risk factors; however, Manish Bansal *et al.* ^29^ found that the FRS cannot be applied efficiently to the Indian population. They did, however, find the JBS3 risk score calculator to be the best predictor of a CV event in high risk Indians. We used the JBS3 risk score to calculate 10-year risk of developing CVD, physiological heart age and life expectancy. Statistically significant data revealed the physiological heart age of the patients to be much higher than their actual age and their life expectancy shortened with more CV risk factors. The shortened life expectancy was much more common in patients with severe OA as they also had a higher JBS3 risk of developing CVD over the next 10 years. It was observed that the mean physiological heart age was as much as 10 years higher than the mean age for the entire study population, drawing a strong correlation between OA and the cardiovascular system.

The mean JBS3 risk was 11% for patients with grade 2 OA, 23% for patients with grade 3 OA and 38% with grade 4 OA. This was along the same lines as studied by Ho Sun Kim *et al.* in Korea, ^11^ where multiple comparisons were made within the different grades of knee OA while studying the relationship with JBS3 risk and all were found to be highly significant. Our study had 225 patients with a prevalence of grade 4 knee OA of 13%. With the help of the ROC curve, the cut-off value of JBS3 was calculated to be 14%, which implies the amount of risk patients with grade 4 knee OA had of developing a CVD in the next 10 years, with a sensitivity of 72.3% and specificity of 66%. It would not be an overstatement to say that a patient with grade 4 knee OA undergoing total knee replacement could have a CV risk of as much as 14%.

India, although one of the countries where both OA and CVD is most prevalent, has very little literature stating the relationship between the two. The strength of this study lies in the different variables (demographic and CV risk factors) that were studied. With the genetic and phenotypic variations in the population in this part of the world, BMI plays a major role in understanding various pathologies in human body. The BG Prasad scale has helped us to divide the study population into various socio-economic strata and study its relation to both OA and CV risk factors. In addition, the diagnosis of knee OA has been made with the help of standard radiographs using K-L classification. The potential confounding factors (CV risk factors) are clubbed together with the help of a standardized risk score calculator devised by the Joint British Society. JBS3 also tells us about the physiological heart age and life expectancy, which may be easier for patients to comprehend. Although these factors have an individual effect on the pathomechanisms of knee OA, when they are used as a part of this scale, various limitations are eliminated, and they are studied as a whole. The risk factors thus are studied both individually as well as co-dependently. However, this study has its limitations too. It is a cross-sectional study, which comes with its own inherent limitations. The study population only has patients with K-L grade of ≥2, so the risk assessment of patients with K-L grade 1 was not done here. The JBS3 risk score calculator used here could only come close to being ideal for the study population of our country. A risk score calculator developed in our country for Indian genotypes and phenotypes would be ideal. Also, it doesn’t include stress as a variable, which has a known effect on cardiovascular status. ^30^ The SBP values entered here are of single reading, which can be affected by the hemodynamic status of the patient at one particular moment. The readings of TC and HDL values may have differed depending on the investigator and equipment available from place to place. Some variables of this tool depend upon the history given by the patient, which has its own limitations. The JBS3 risk score calculator is not a tool to initiate treatment for a patient, rather a tool that gives a fair idea of the patient’s general condition and CV status.

## Conclusion

Our study concluded that there is a strong positive correlation between knee OA and CVD with CV risk score being directly proportional to the severity of OA. JBS3 is a comprehensive risk score calculator as well as a screening tool that produces three more variables, namely 10-year risk of developing CVD, physiological heart age and life expectancy, which are easy to comprehend.

### Implication

The JBS3 risk score calculator, a comprehensive tool, can be applied to the general population in routine clinical practice to comprehensively address the CV risk factors in patients with knee OA. At the same time, screening for the same risk factors can be carried out in unaware high-risk patients. A population-based study with similar tools can help us derive a JBS3 risk score cut-off, which would aid in management of patients undergoing total knee replacement. The JBS3 risk score calculator/mobile-based application could help health care professionals to better illustrate the risk of CVD and the gains that can be made by early interventions.

## Data availability

### Underlying data

Dryad: Assessment of cardiovascular risk factors in patients with Knee Osteoarthritis. https://doi.org/10.5061/dryad.79cnp5htv. ^30^

Data are available under the terms of the Creative Commons Zero “No rights reserved” data waiver (CC0 1.0 Public domain dedication).
