# Supplementary material for: Cross-sectional assessment of cardiovascular risk factors in patients with knee osteoarthritis
Source: F1000Res. 2022 Feb 17;10:508. Originally published 2021 Jun 28. [Version 3] doi: 10.12688/f1000research.27744.3 (PMC8866909; doi:10.12688/f1000research.27744.3)
Supplement: Peer review history

# Peer review: Reviewer response for version 3

**DOI:** 10.5256/f1000research.121262.r124117

**Published:** 2022-02-23

**Authors:** Hayden F Atkinson, Jenna Schulz

**Affiliations:** 1 University of Prince Edward Island, Charlottetown, C1A 4P3, Canada; 2 Western University, London, ON, Canada

We have reviewed the authors' responses to our suggestions, as well as the changes made to the manuscript. We are satisfied with the responses and the newest version of the manuscript. We are happy to approve the manuscript for indexing with no reservations.

Is the work clearly and accurately presented and does it cite the current literature?

Partly

If applicable, is the statistical analysis and its interpretation appropriate?

Partly

Are all the source data underlying the results available to ensure full reproducibility?

Yes

Is the study design appropriate and is the work technically sound?

Partly

Are the conclusions drawn adequately supported by the results?

Partly

Are sufficient details of methods and analysis provided to allow replication by others?

Yes

Reviewer Expertise:

NA

We confirm that we have read this submission and believe that we have an appropriate level of expertise to confirm that it is of an acceptable scientific standard.

# Peer review: Reviewer response for version 2

**DOI:** 10.5256/f1000research.79143.r119851

**Published:** 2022-02-08

**Authors:** Hayden F Atkinson, Jenna Schulz

**Affiliations:** 1 University of Prince Edward Island, Charlottetown, C1A 4P3, Canada; 2 Western University, London, ON, Canada

Thank you for the invitation to review this manuscript. The authors examined cardiovascular (CV) risk factors in patients with knee osteoarthritis (OA), and concluded that there is a strong correlation between OA and CV disease, with the severity of OA and CV risk score being directly proportional.

 Overall, this paper is well written, but there is some room for improvement. Specifically, the discussion would benefit from being more concise, and an improved overall flow to help tell the story the authors are trying to get across.

**Abstract**

- The second methods sentence is quite long. Suggestion to break it up into two smaller sentences.
- Please correct all instances in the abstract and text stating that CV risk score is directly proportional to the severity of knee OA and replace with “positively correlated”. ‘Directly proportional’ infers that the correlation is perfect.

**Introduction**

- Second paragraph: “OA is associated with knee pain”. Suggested to revise to “joint” pain as OA can occur in any synovial joint. Other option is to introduce the focus specifically as knee OA in the first paragraph.
- Third paragraph: If the authors could avoid namedropping as they have done in the following example and cite the source as they have done in the remainder of the text it would make for easier reading: “Dr. J.S. Lawrence [and his] work at Leigh where he…”. Same with the paragraph in the methods section with many names mentioned after the sample size calculation, and several other instances throughout the text.
- Third paragraph: It is recommended the authors cite the systematic review by Schulz *et al*., 2020 ^1^ at the end of this paragraph as a very relevant potential source of increased risk for CV disease in OA patients is the under-prescription of aerobic exercise for patients with knee OA. Health care providers often under-prescribe exercise, especially aerobic exercise for patients with osteoarthritis. Exercise physiologists agree that 150 minutes of moderate-to-vigorous physical activity on weekly basis contributes to reducing chronic disease risk and severity, especially for cardiovascular disease. Patients with OA should be instructed to meet these guidelines, however, research suggests that health care providers are prescribing exercise volumes below this 150-minute threshold, which can contribute to increased risk for CV disease onset or progression in patients with OA.
- Suggestion to add the concept of “systemic inflammation” as potential pathogenesis underlying both OA and CVD. Although not a primarily inflammatory disease, there is now evidence of a systemic component to OA, as opposed to the traditional view of a “wear and tear” disease.

**Study Participants**

- State that you did not exclude based on other comorbidities/systemic diseases (i.e. diabetes, hypertension, RA, etc). Including these patients could potentially confound results and therefore cannot draw causative conclusions (i.e. knee OA causes CVD or vice versa), and should make sure to keep results purely correlative in discussion/conclusion.
- Please provide an explanation as to why patients with secondary knee OA were not eligible to participate.

**Statistical analysis**

- Pearson correlation between JBS3 score and KL Grade is inappropriate as KL Grade is a categorical variable and not a quantitative variable (no direct, linear relationship between Grade 1-2-3-4). Pearson correlations are conducted between two continuous variables. All mentions of a Pearson correlation between KL Grade and JBS3 score will have to be removed. Please choose an appropriate analysis that permits correlation between a categorical variable and a continuous variable. Results will likely be similar but please ensure the test chosen is appropriate. The article by Gupta, 1960 ^2^ may be helpful.
- Rheumatoid arthritis was mentioned in abbreviated form earlier (under variables).

**Results**

- Suggestion to remove the “inference” column to clean up the results table and just state in results that a p-value of <0.05 was deemed significant.
- Figure 5: Suggest removing the figure and replacing it with a table or just words in discussion.

**Discussion**

- Suggestion to begin the discussion and each paragraph by summarizing key findings from results, then using evidence from recent papers to support results.
- The paragraphs relating to age can likely be condensed into one.
- The paragraph relating to OA and socioeconomic status should also be clarified and condensed. Suggestion to remove the sentence and citation of the study in Japan and a higher prevalence of OA in mountainous regions as there is no mention in this current study about where participants were from or their current activity status. Additionally, OA is multifactorial (as listed in the sentence below), and attributing the cause of OA to activity (when we know inactivity is a large risk factor) is a bit of an over-conclusion.
- Suggestion to remove “many doctors and researchers who believe that…” and condense to the “association between knee OA and CVD has been studied and linked to underlying systemic inflammation, however, it is unsure if the relationship is either direct or indirect. For example…” and cite studies. The details of these studies (i.e. number of participants, exact results) could be removed and this would help condense the paragraph down.
- Suggestion to remove the sentence regarding the conclusion that patients with severe knee OA have a higher risk of developing CVD in the future as this is an over-drawn conclusion. Instead, rephrase to discuss the correlation between knee OA and risk factors for CVD found in this study.
- Suggestion to flip the order of the last two sentences in this paragraph.
- Suggestion to replace assumption with hypothesis.
- Suggestion to remove the information about the FRS to make it more concise and condense this paragraph with the following.
- Are the risk factors in the JBS3 score made to be studied individually or meant to be kept together as a whole? Please expand on this and add it as a limitation to the study. Also, suggest adding a limitation surrounding that there was no information regarding activity levels in the study participants.

**Conclusion/implication**

- There was no mention in the introduction of this study being used to determine the feasibility of implementing the JBS3 score. Refer back to objectives, and reflect on those here. Suggestion to keep the conclusion/implication section around the correlation between knee OA and CVD, and how these findings may impact health care practitioners, patients, and the field as a whole.

Is the work clearly and accurately presented and does it cite the current literature?

Partly

If applicable, is the statistical analysis and its interpretation appropriate?

Partly

Are all the source data underlying the results available to ensure full reproducibility?

Yes

Is the study design appropriate and is the work technically sound?

Partly

Are the conclusions drawn adequately supported by the results?

Partly

Are sufficient details of methods and analysis provided to allow replication by others?

Yes

Reviewer Expertise:

NA

We confirm that we have read this submission and believe that we have an appropriate level of expertise to confirm that it is of an acceptable scientific standard, however we have significant reservations, as outlined above.

## References

[1] : Are we missing the target? Are we aiming too low? What are the aerobic exercise prescriptions and their effects on markers of cardiovascular health and systemic inflammation in patients with knee osteoarthritis? A systematic review and meta-analysis. *Br J Sports Med* .2020;54(13) : 10.1136/bjsports-2018-100231 771-775 10.1136/bjsports-2018-100231 31848152

[2] : Point biserial correlation coefficient and its generalization. *Psychometrika* .1960;25(4) : 10.1007/BF02289756 393-408 10.1007/BF02289756

# Response

**Published:** 2022-02-18

**Authors:** Sagar Goel

**Affiliations:** MAHE, MANIPAL, India

Thank you for your valuable feedback which has helped us to improve this paper. We have attempted to address all your suggestions and queries as follows:

**Abstract**

- The second methods sentence is quite long. Suggestion to break it up into two smaller sentences.

**Author response:** Done.
- Please correct all instances in the abstract and text stating that CV risk score is directly proportional to the severity of knee OA and replace with “positively correlated”. ‘Directly proportional’ infers that the correlation is perfect.

**Author response:** Done.

**Introduction**

- Second paragraph: “OA is associated with knee pain”. Suggested to revise to “joint” pain as OA can occur in any synovial joint. Other option is to introduce the focus specifically as knee OA in the first paragraph.

**Author response:** Done.
- Third paragraph: If the authors could avoid namedropping as they have done in the following example and cite the source as they have done in the remainder of the text it would make for easier reading: “Dr. J.S. Lawrence [and his] work at Leigh where he…”. Same with the paragraph in the methods section with many names mentioned after the sample size calculation, and several other instances throughout the text.

**Author response:** Done.
- Third paragraph: It is recommended the authors cite the systematic review by Schulz *et al*., 2020 ^1^ at the end of this paragraph as a very relevant potential source of increased risk for CV disease in OA patients is the under-prescription of aerobic exercise for patients with knee OA. Health care providers often under-prescribe exercise, especially aerobic exercise for patients with osteoarthritis. Exercise physiologists agree that 150 minutes of moderate-to-vigorous physical activity on weekly basis contributes to reducing chronic disease risk and severity, especially for cardiovascular disease. Patients with OA should be instructed to meet these guidelines, however, research suggests that health care providers are prescribing exercise volumes below this 150-minute threshold, which can contribute to increased risk for CV disease onset or progression in patients with OA.

**Author response:** Done.
- Suggestion to add the concept of “systemic inflammation” as potential pathogenesis underlying both OA and CVD. Although not a primarily inflammatory disease, there is now evidence of a systemic component to OA, as opposed to the traditional view of a “wear and tear” disease.

**Author response:** Done.

**Study Participants**

- State that you did not exclude based on other comorbidities/systemic diseases (i.e. diabetes, hypertension, RA, etc). Including these patients could potentially confound results and therefore cannot draw causative conclusions (i.e. knee OA causes CVD or vice versa), and should make sure to keep results purely correlative in discussion/conclusion.

**Author response:** The exclusion criteria mention only patients with cardiovascular disease which we find is self-explanatory that all the other comorbid conditions were not excluded.
- Please provide an explanation as to why patients with secondary knee OA were not eligible to participate.
-
**Author response:** Our goal was to find an association between primary OA and CV risk factors that's why secondary OA was excluded.

**Statistical analysis**

- Pearson correlation between JBS3 score and KL Grade is inappropriate as KL Grade is a categorical variable and not a quantitative variable (no direct, linear relationship between Grade 1-2-3-4). Pearson correlations are conducted between two continuous variables. All mentions of a Pearson correlation between KL Grade and JBS3 score will have to be removed. Please choose an appropriate analysis that permits correlation between a categorical variable and a continuous variable. Results will likely be similar but please ensure the test chosen is appropriate. The article by Gupta, 1960 ^2^ may be helpful.

**Author response:** We have decided to remove Pearson's correlation as this was wrongly used by us. We have not tried to add any other method as it was not providing any additional information pertaining to the results.
- Rheumatoid arthritis was mentioned in abbreviated form earlier (under variables).

**Author response:** Done.

**Results**

- Suggestion to remove the “inference” column to clean up the results table and just state in results that a p-value of <0.05 was deemed significant.

**Author response:** The inference column has been removed.
- Figure 5: Suggest removing the figure and replacing it with a table or just words in discussion.

**Author response:** Done.

**Discussion**

- Suggestion to begin the discussion and each paragraph by summarizing key findings from results, then using evidence from recent papers to support results

**Author response:** Done.
- The paragraphs relating to age can likely be condensed into one.

**Author response:** Done.
- The paragraph relating to OA and socioeconomic status should also be clarified and condensed. Suggestion to remove the sentence and citation of the study in Japan and a higher prevalence of OA in mountainous regions as there is no mention in this current study about where participants were from or their current activity status. Additionally, OA is multifactorial (as listed in the sentence below), and attributing the cause of OA to activity (when we know inactivity is a large risk factor) is a bit of an over-conclusion.

**Author response:** Done.
- Suggestion to remove “many doctors and researchers who believe that…” and condense to the “association between knee OA and CVD has been studied and linked to underlying systemic inflammation, however, it is unsure if the relationship is either direct or indirect. For example…” and cite studies. The details of these studies (i.e. number of participants, exact results) could be removed and this would help condense the paragraph down.

**Author response:** Done.
- Suggestion to remove the sentence regarding the conclusion that patients with severe knee OA have a higher risk of developing CVD in the future as this is an over-drawn conclusion. Instead, rephrase to discuss the correlation between knee OA and risk factors for CVD found in this study.

**Author response:** Done.
- Suggestion to flip the order of the last two sentences in this paragraph.

**Author response:** Done.
- Suggestion to replace assumption with hypothesis.

**Author response:** Done.
- Suggestion to remove the information about the FRS to make it more concise and condense this paragraph with the following.

**Author response:** Done.
- Are the risk factors in the JBS3 score made to be studied individually or meant to be kept together as a whole? Please expand on this and add it as a limitation to the study.

**Author response:** The CV risk factors were analysed individually as well as whole using the JBS3 risk score calculator as evident in the results and discussion part.
- Also, suggest adding a limitation surrounding that there was no information regarding activity levels in the study participants.

**Author response:** Done.

**Conclusion/implication**

- There was no mention in the introduction of this study being used to determine the feasibility of implementing the JBS3 score. Refer back to objectives, and reflect on those here. Suggestion to keep the conclusion/implication section around the correlation between knee OA and CVD, and how these findings may impact health care practitioners, patients, and the field as a whole.

**Author response:** Thank you for your suggestion and the conclusion and implications have been rephrased.

# Peer review: Reviewer response for version 2

**DOI:** 10.5256/f1000research.79143.r101124

**Published:** 2021-12-07

**Authors:** Win Min Oo, Htoo Dar Li

**Affiliations:** 1 Rheumatology Department, Royal North Shore Hospital, Institute of Bone and Joint Research, Kolling Institute, Faculty of Medicine and Health, The University of Sydney, Sydney, NSW, Australia; 2 University of Medicine, Mandalay, Myanmar

We have reviewed the manuscript and found that the authors have already responded to most of our concerns satisfactorily.

Is the work clearly and accurately presented and does it cite the current literature?

Partly

If applicable, is the statistical analysis and its interpretation appropriate?

Partly

Are all the source data underlying the results available to ensure full reproducibility?

Yes

Is the study design appropriate and is the work technically sound?

Partly

Are the conclusions drawn adequately supported by the results?

Partly

Are sufficient details of methods and analysis provided to allow replication by others?

Yes

Reviewer Expertise:

NA

We confirm that we have read this submission and believe that we have an appropriate level of expertise to confirm that it is of an acceptable scientific standard.

# Peer review: Reviewer response for version 1

**DOI:** 10.5256/f1000research.30677.r92755

**Published:** 2021-09-09

**Authors:** Win Min Oo, Htoo Dar Li

**Affiliations:** 1 Rheumatology Department, Royal North Shore Hospital, Institute of Bone and Joint Research, Kolling Institute, Faculty of Medicine and Health, The University of Sydney, Sydney, NSW, Australia; 2 University of Medicine, Mandalay, Myanmar

It’s been a great pleasure reviewing this manuscript. The authors examined various cardiovascular (CV) risk factors in 225 patients with knee OA, concluding that a strong positive correlation between knee OA and CVD existed.

 Generally, the manuscript is well written though there is still a large space for much improvement. I have several comments to improve it.

**Abstract:**

- In the conclusion section, we would suggest omitting some sentences as it is not appropriate to mention them here. Perhaps the best place would be in the methodology as the outcome measure: "JBS3 is a comprehensive risk score calculator as well as a screening tool, which…".

**Introduction:**

- Third paragraph: Generally, systemic inflammation is not part of the OA disease process nor florid as in rheumatoid arthritis which has an obvious association with CVD.
- References 3 and 4 you used for OA being associated with high cholesterol levels did not mention such - page 3, third paragraph: ”recent studies have reported that OA is associated with high serum cholesterol levels in females”.

**Methodology:**

*Study design*

- Do you mean that a convenience sample was used? Any retrospective data? If so, please mention it.

*Study participants*

- Just wondering why the American College of Rheumatology criteria was not used for case definition. How are references 9 and 10 appropriate here, especially for case definition?
- For the sample size calculation, the estimated proportion was taken from a Korean study in which the prevalence of knee OA may be different from an Indian population, and it may be overestimated as the study was not conducted in the community setting.
- Are there any Indian studies for the record of community prevalence of knee OA? We think it would be more relevant if available.

*Variables*

- In knee X-rays, did you consider only patellofemoral or tibiofemoral OA or both eligible?
- Physical inactivity (sedentary lifestyle) and NSAID usage should be included and adjusted as the potential confounders.

*Statistical analysis*

- Just wondering why the multiple regression model for adjusting the confounders was not used.
- For post-hoc analysis, did you conduct any correction of the p-value for multiple comparisons? If so, please mention it here.

**Results**

- Instead of using many figures for each of the baseline characteristics, one table should work well for all variables. Strongly suggest doing so.
- Table 2 is difficult to understand without the inputs of blood pressure calculated. Suggested to modify it.
- Too many figures and interpretations for a research article. Please be concise.
- The current or previous medication used for OA should be reported as NSAID especially Cox-2 inhibitors may increase the CVD risk and morbidity. Therefore, such confounders should be adjusted in the statistical analysis to draw a valid interpretation.

**Discussion**

- Too long and involves the repetition of much of the results section instead of correlating with the findings of other papers.
- The redundancy of the language should be avoided, i.e. "This is the first study of its type to be conducted in this part of the world. India...".

Is the work clearly and accurately presented and does it cite the current literature?

Partly

If applicable, is the statistical analysis and its interpretation appropriate?

Partly

Are all the source data underlying the results available to ensure full reproducibility?

Yes

Is the study design appropriate and is the work technically sound?

Partly

Are the conclusions drawn adequately supported by the results?

Partly

Are sufficient details of methods and analysis provided to allow replication by others?

Yes

Reviewer Expertise:

Osteoarthritis; Musculoskeletal imaging; Neurorehabilitation

We confirm that we have read this submission and believe that we have an appropriate level of expertise to confirm that it is of an acceptable scientific standard, however we have significant reservations, as outlined above.

# Response

**Published:** 2021-11-04

**Authors:** Sagar Goel

**Affiliations:** MAHE, MANIPAL, India

Thank you for your feedback. We have tried our best to respond to some of your queries and made some modifications to the manuscript as per your suggestions. 

**Abstract:**

- In the conclusion section, we would suggest omitting some sentences as it is not appropriate to mention them here. Perhaps the best place would be in the methodology as the outcome measure: "JBS3 is a comprehensive risk score calculator as well as a screening tool, which…".

**Author response:** Done.

**Introduction:**

- Third paragraph: Generally, systemic inflammation is not part of the OA disease process nor florid as in rheumatoid arthritis which has an obvious association with CVD.

**Author response:** Done.
- References 3 and 4 you used for OA being associated with high cholesterol levels did not mention such - page 3, third paragraph: ”recent studies have reported that OA is associated with high serum cholesterol levels in females”.

**Author response:** The text has been modified and the concerned reference is added.

**Methodology:**

*Study design*

- Do you mean that a convenience sample was used? Any retrospective data? If so, please mention it. 

**Author response:** Yes, a convenience sample was used. Moreover, retrospective data was also collected as mentioned under the heading Ethics Statement and Sampling: “Also, case records of patients diagnosed with knee OA between January 2017 to September 2018 were studied…”

*Study participants*

- Just wondering why the American College of Rheumatology criteria was not used for case definition. How are references 9 and 10 appropriate here, especially for case definition?

**Author response:** We believed Kellgren and Lawrence grading of OA knee based on the X-ray was more suitable for case definition here. Using reference no 9, we decided to keep 50 years of age as a cutoff for patient selection.

- For the sample size calculation, the estimated proportion was taken from a Korean study in which the prevalence of knee OA may be different from an Indian population, and it may be overestimated as the study was not conducted in the community setting.
- Are there any Indian studies for the record of community prevalence of knee OA? We think it would be more relevant if available.

**Author response:** The Prevalence of knee OA among Indian and Korean Populations is comparable and has been mentioned with reference

*Variables*

- In knee X-rays, did you consider only patellofemoral or tibiofemoral OA or both eligible?

**Author response:** Both were considered.

- Physical inactivity (sedentary lifestyle) and NSAID usage should be included and adjusted as the potential confounders.

**Author response:** We used the JBS3 risk score calculator which did not include physical inactivity and NSAID usage as variables.

*Statistical analysis*

- Just wondering why the multiple regression model for adjusting the confounders was not used.

**Author response:** Done and added.
- For post-hoc analysis, did you conduct any correction of the p-value for multiple comparisons? If so, please mention it here.

**Author response:** Yes, it was done.

**Results**

- Instead of using many figures for each of the baseline characteristics, one table should work well for all variables. Strongly suggest doing so.

**Author response:** Done.
- Table 2 is difficult to understand without the inputs of blood pressure calculated. Suggested to modify it.

**Author response:** Done.
- Too many figures and interpretations for a research article. Please be concise.

**Author response:** Done.
- The current or previous medication used for OA should be reported as NSAID especially Cox-2 inhibitors may increase the CVD risk and morbidity. Therefore, such confounders should be adjusted in the statistical analysis to draw a valid interpretation.

**Author response:** We used the JBS3 risk score calculator which did not include physical inactivity and NSAID usage as variables.

**Discussion**

- Too long and involves the repetition of much of the results section instead of correlating with the findings of other papers.

**Author response:** Done.
- The redundancy of the language should be avoided, i.e. "This is the first study of its type to be conducted in this part of the world. India...". 

**Author response:** Done.
